# Frequency and patterns of CMF emergency cases during and after COVID-19

**DOI:** 10.1007/s00068-025-02957-w

**Published:** 2025-09-03

**Authors:** Philipp Thoenissen, Leonie Ditt, Philip Terwey, Maximilian Leiblein, Shahram Ghanaati, Robert Sader, Benjamin Friedrichson

**Affiliations:** 1 Department of Oral, Cranio-Maxillofacial and Plastic Facial Surgery, Goethe University Frankfurt, University Hospital, Theodor-Stern-Kai 7, 60590 Frankfurt am Main, Germany; 2 Department of Anesthesiology, Intensive Care Medicine and Pain Therapy, Goethe University Frankfurt, University Hospital, Theodor-Stern-Kai 7, 60590 Frankfurt am Main, Germany; 3 Department of Trauma, Hand and Reconstructive Surgery, Goethe University Frankfurt, University Hospital, Theodor-Stern-Kai 7, 60590 Frankfurt am Main, Germany

**Keywords:** Craniomaxillofacial trauma, COVID-19, Facial fractures, Public health, ICD-10, Trauma epidemiology

## Abstract

**Background:**

Craniomaxillofacial (CMF) trauma constitutes a significant proportion of hospital presentations, often resulting from high-energy mechanisms such as interpersonal violence and traffic accidents. The COVID-19 pandemic and associated public health restrictions markedly altered daily life and social behavior, potentially influencing trauma patterns and emergency healthcare utilization.

**Methods:**

We conducted a retrospective analysis of nationwide anonymized inpatient data from German hospitals, reported to the National Institute for the Hospital Remuneration System (InEK), covering the period from March 18, 2019 to March 17, 2023. CMF trauma cases were identified using ICD-10 codes and stratified across four timeframes: pre-pandemic, pandemic, post-pandemic, and normalization. Statistical analysis included descriptive evaluation and Poisson regression.

**Results:**

A total of 118,620 CMF-related diagnoses were recorded in the pre-pandemic period, which declined significantly during the pandemic (− 14.66%, *p* < 0.0001). Although case numbers increased in the post-pandemic (+ 4.95%, *p* < 0.0001) and normalization periods (+ 12.65%, *p* < 0.0001 compared to pandemic), they did not fully return to pre-pandemic levels. The largest relative declines were observed for mandibular and midfacial fractures. In contrast, general trauma indicators such as distal radius fractures remained relatively stable, suggesting a trauma-mechanism-specific effect.

**Conclusion:**

The COVID-19 pandemic significantly reduced the number of CMF emergency cases in Germany, with partial recovery observed in subsequent years. These findings reflect shifts in trauma etiology and healthcare-seeking behavior during and after pandemic-related societal changes.

## Introduction

In Germany, emergency units provide capacity for several entities of traumatological care as a subdivision of hospitals. Craniomaxillofacial (CMF) injuries are common and are challenging to traumatological teams. Patients with CMF injuries have a high preclinical and clinical mortality, as well as a high risk to be misdiagnosed [1, 2]. These injuries often result from high energy trauma like assaults and traffic accidents which are related to daily routine life. Emergency units provide access to in-hospital care. Inpatient care followed by surgery is one treatment of choice and can be conducted only in specialized centers. After the occurrence of the COVID-19 the daily routine life has changed. In consequence, it needs to be questioned to which amount the frequency of CMF trauma was influenced by the COVID-19 and pandemic related governmental restrictions and how these changes were affecting the rare capacity of CMF care.

Therefore this study analyzed German governmental data on common CMF trauma according to International classification of diseases (ICD)−10 for a pre-, a pandemic, a post-pandemic and normalization period.

## Materials and methods

### Data acquisition

The retrospective data collection was performed by accessing performance data from all German hospitals provided by the National Institute for the Hospital Remuneration System (Institut für das Entgeltsystem im Krankenhaus GmbH, InEK). In Germany, hospitals are required by law to report data of all inpatients in an anonymized form to InEK (§ 21 KHEntgG). This data is collected with the purpose of continuously developing the existing reimbursement system, which is based on diagnosis related groups (DRG). Since 2020, open access to these data is possible. Datasets are anonymized and are open for scientific research according to German law. Therefore, the Ethics Committee of the University Hospital Frankfurt waived the requirement for Ethics Committee approval for this study (Ref. 2022 − 766).

Case presentations were identified via a retrospective systematical query in the database using the International Statistical Classification of Diseases and Related Health Problems Version 10 (ICD-10) codes for CMF diagnoses of the German Diagnosis Related Groups (G-DRG) and certain OPS-Codes. Admission information obtained included age and sex.

Absolute (total) numbers have been evaluated by summing main and secondary diagnoses which were documented within the stay as inpatient in hospital. In this study, the term “all diagnoses” refers to the total number of cases derived from summing the main diagnoses (MD) and the secondary diagnoses (SD) documented during each inpatient stay.

The results part lists the sum of main diagnoses (MD) and secondary diagnoses (SD) and also entails a separation between those two categories. Due to data protection regulations, a more detailed query at the individual patient level is not possible.

The data presented originate exclusively from German hospitals involved in inpatient care. The database does not specify which clinical department (e.g., general surgery, trauma surgery, orthopedics, craniomaxillofacial surgery, or internal medicine) was responsible for the documentation.

### COVID-19-related time periods

The study period ranged from 03/18/2019 to 03/17/2023 and was divided into four groups: a pre-pandemic cohort (03/18/2019–03/17/2020), a pandemic cohort (03/18/2020–03/17/2021), a post-pandemic cohort (03/18/2021–03/17/2022) and a cohort that reflects normality (03/18/2022–03/17/2022). The cut-off date of 17 March 2020 was selected based on the initiation of the national hospital emergency plan by the German Government to address the pandemic.

### ICD-10 codes of interest

**Fig. 1:**
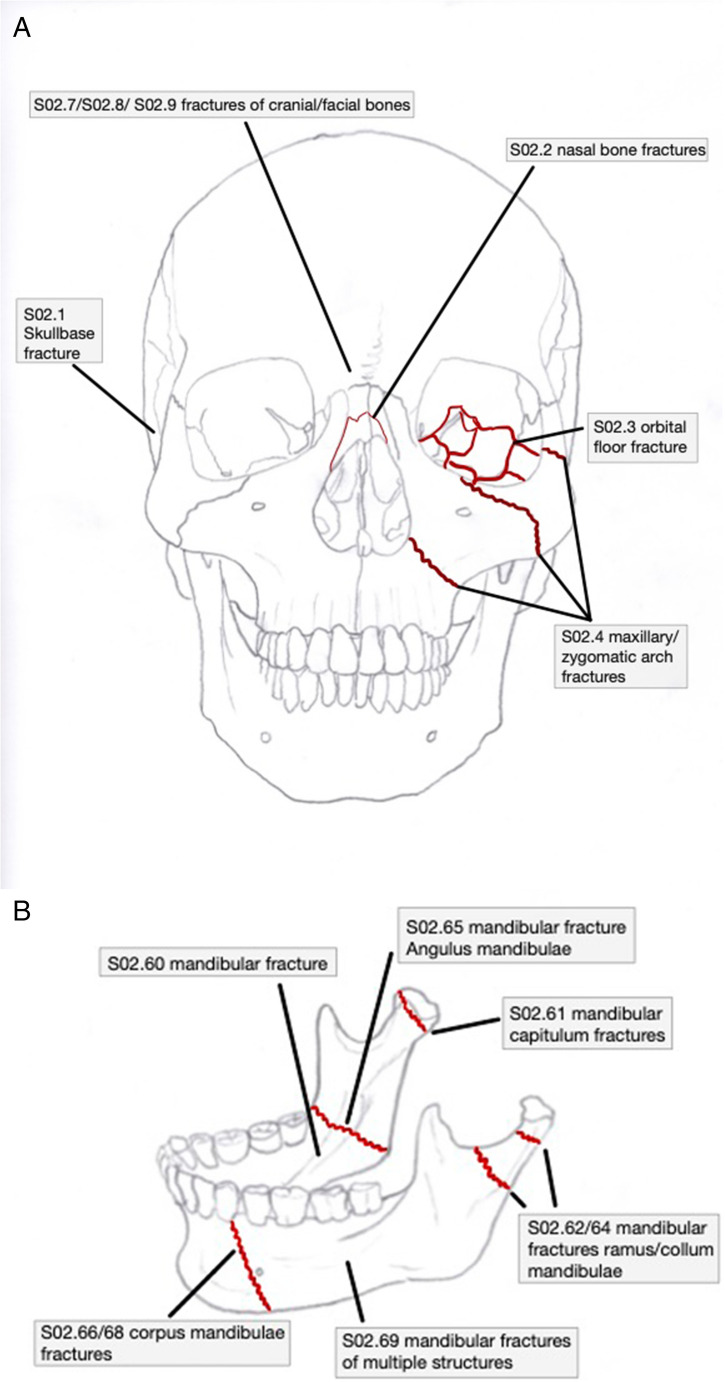
Depiction of the common ICD-10-Codes to the midface and skull (**A**) and mandible (**B**)

The particular ICD-10 codes of interest list fractures related to the mandible, midface and cranial structures (Fig. 1A and B). The following fractures were set for analysing trauma of the mandible: S.02.60 (mandibular fractures), S02.61 (capitulum fracture), S02.62 and S02.64 (collum fractures), S02.65 (angulus mandibulae), S02.66 and S02.68 (corpus or symphysis mandibulae), S02.63 (proc. coronoideus) and S02.69 (multiple mandibular fractures).

The following codes were set for the analysis of trauma associated with midfacial, nasal bone structures, the orbital floor, maxillary bone and zygomatic arch: S02.2, S02.3 and S02.4, respectively. For analysing skull base fractures the following codes were set: S02.1. Further fractures of cranial bones or facial bones are coded by S02.7, S02.8 and S02.9.

According to the ICD-10-classification, the cases were allocated to a pre-pandemic, pandemic, a post-pandemic and normality group.

#### Statistical analysis

Statistical analysis was performed using Excel and Prism Graphpad using a Poisson-Regression Test. *P*-values < 0.05 were considered to be significant. The diagnoses were collected and descriptively and exploratively characterized and analyzed. Next to purely descriptive methods, time series statistics and periodical day influences were analyzed.

## Results

### General alterations

The study included all patients with CMF injuries, who were admitted to German hospitals in a pre-pandemic period from 03/18/2019 to 03/17/2020, a pandemic period from 03/18/2020 to 03/17/2021, a post-pandemic period from 03/18/2021 to 03/17/2022 and a period of normality 02/18/2022 to 03/17/2023.

Every day, a total of 312 fractures are diagnosed in oral and maxillofacial surgery in Germany. Of these, 38 mandibular fractures are diagnosed, 214 midface fractures and 60 skull fractures. During the pandemic period, the number of diagnosed fractures per day in Germany has fallen to a total of 277 fractures in oral and maxillofacial surgery. Of these, 32 mandibular fractures are diagnosed per day, 188 midface fractures and 56 skull fractures.

Focusing on the main diagnoses, 36.368 CMF patients were identified in the pre-pandemic period. 64.58% were male (*n* = 23.485), 35.41% were female (*n* = 12.877); in 0,02% (*n* = 6) sex was unknown. In the pandemic period, a total number of 29.017 CMF patients was identified. 62.15% were male (*n* = 18.034), 37.84% were female (*n* = 10.980), 0.01% diverse (*n* = 2), 1 patient of unknown gender. The overall decrease of CMF related presentations is 20.21% (*p* < 0.0001) compared to the pre-pandemic period. In the post-pandemic period, a total number of 31.020 CMF patients was identified. 64.58% were male (*n* = 20.033), 35.4% were female (*n* = 10.982) and 0.02% (*n* = 5) were unknown. The overall increase of CMF related presentations was 6.9% (*p* < 0.0001) in comparison to the pandemic period. In comparison between the pre-pandemic and post-pandemic period, a decrease of 14.71% (*p* < 0.0001) was recorded.

After normalizing, a total number of 32.893 CMF patients was identified. 64.47% were male (*n* = 21.207), 35.51% were female (*n* = 11.679), 0.01% were divers (*n* = 4) and 0.01% (*n* = 3) were patients of unknown gender. The overall increase of CMF related presentations between the pandemic period and normality was 13.36% (*p* < 0.0001). In comparison between pre-pandemic to normality a decrease of 9,56% (*p* < 0.0001) occurred.

The total amount of all diagnoses is 118.620 diagnoses including MD and SD in the pre-pandemic (36.386 MD, 82.252 SD) and 101.230 diagnoses pandemic (29.017 MD, 72.213 SD) which corresponds to a decrease of 14.66% (*p* < 0.0001). During the post-pandemic period the total amount of 106.238 diagnoses (31.020 MD, 75.218 SD) is noted (+ 4.95%, *p* < 0.0001). In comparison between the pre-pandemic and post-pandemic period, a decrease of 10.44% (*p* < 0.0001) occurred.

**Fig. 2:**
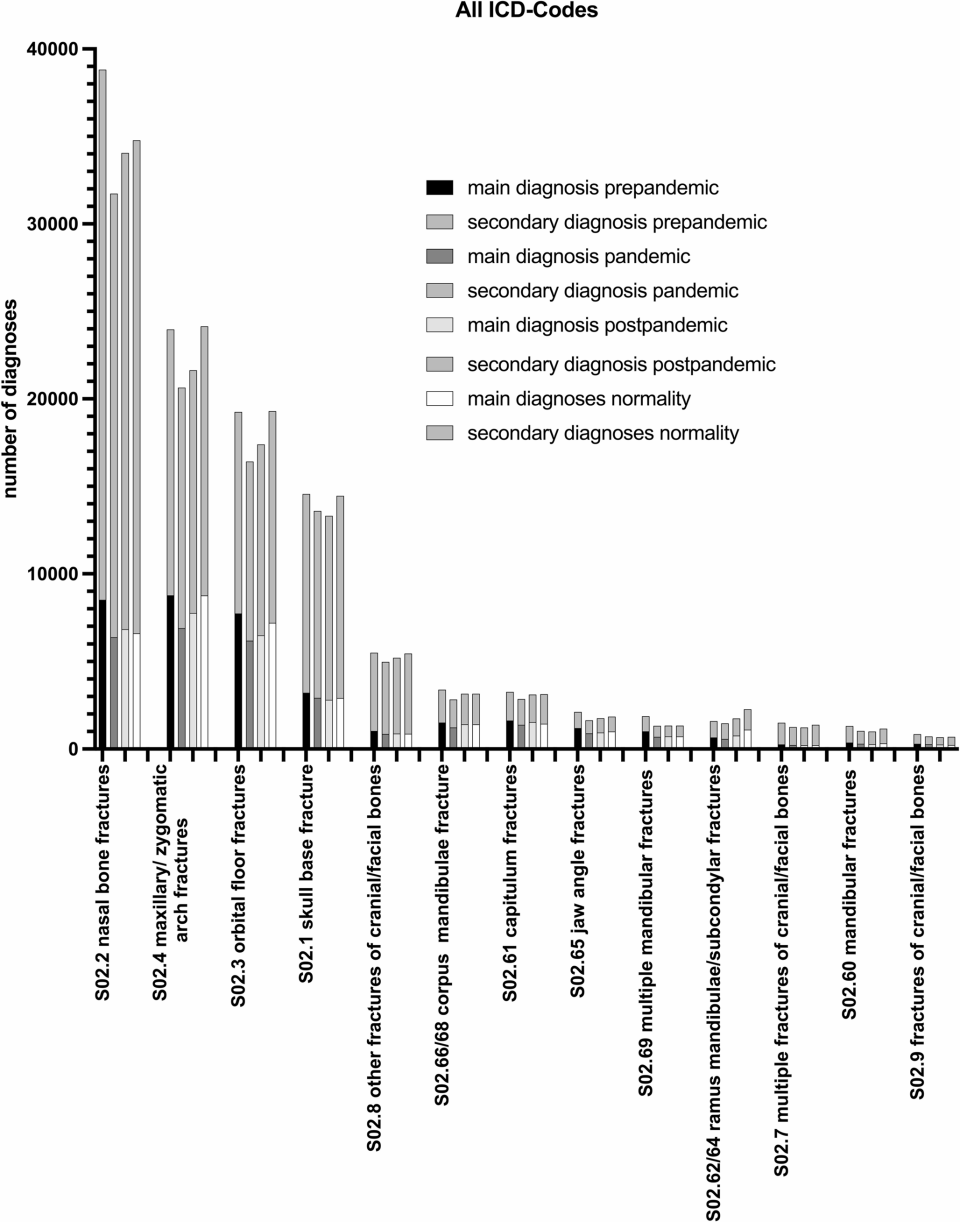
Number of all ICD-10 Codes of interest as inpatients in the pre-pandemic, pandemic, post-pandemic period and normality

During normality the total amount of 114.039 diagnoses (32.928 MD, 81.111 SD) is noted. Comparing the pre-pandemic period to normality, a decrease of 3.86% (*p* < 0.0001) was noted. In comparison of the pandemic to normality, an increase of 12.65% (*p* < 0.0001) and in comparison of post-pandemic to normality also an increase of 7.34% (*p* < 0.0001) occurred. Figure 2 shows the number of diagnoses for the pre-, pandemic, post-pandemic period and normality for all origins of interest (Fig. 2).

In addition to the CMF fractures, evaluation of radius fractures have been performed as it is the most common fracture in general trauma surgery. During the pre-pandemic period, 118.349 radial fractures (MD and SD) were recorded in Germany; and 118.780 fractures during the pandemic period. This corresponds to an increase of 0.36% (*p* = 0.3761). During the post-pandemic period, 117.502 radial fractures were recorded, which represents a decrease of 0.72% (*p* = 0.0811) compared to the pre-pandemic period and 1.1% (*p* = 0.0086) compared to the pandemic period. During normality, 121.215 radial fractures were recorded in Germany, which corresponds to an increase of 2.0–3.16%, respectively (Table 1).

**Table 1:** List of the general alterations for the main diagnoses, all diagnoses and fractures of the radius for the different time periods: pre-pandemic (pre), pandemic (pan), post-pandemic (post), normality (norm). Total numbers are given for the periods, percentage-based comparisons were conducted to evaluate changes across the respective groups (i.e. pre-pan, etc.)

	period	Total number	pre-pan (%)	pan-post (%)	pre-post (%)	pan-nor (%)	pre-nor (%)	post-nor (%)
Main diagnoses	Pre-pandemic	36.368						
	Pandemic	29.017	−20.21					
	Post-pandemic	31.020		6.90	−14.71			
	Normality	32.893				13.36	−9.56	6.04
All	Pre-pandemic	118.620						
	Pandemic	101.230	−14.66					
	Post-pandemic	106.238		4.95	−10.44			
	Normality	114.039				12.65	−3.86	7.34
Radius	Pre-pandemic	118.349						
	Pandemic	118.780	0.36					
	Post-pandemic	117.502		−1.08	−0.72			
	Normality	121.215				2.05	2.42	3.16

### Mandibular fractures

**Fig. 3:**
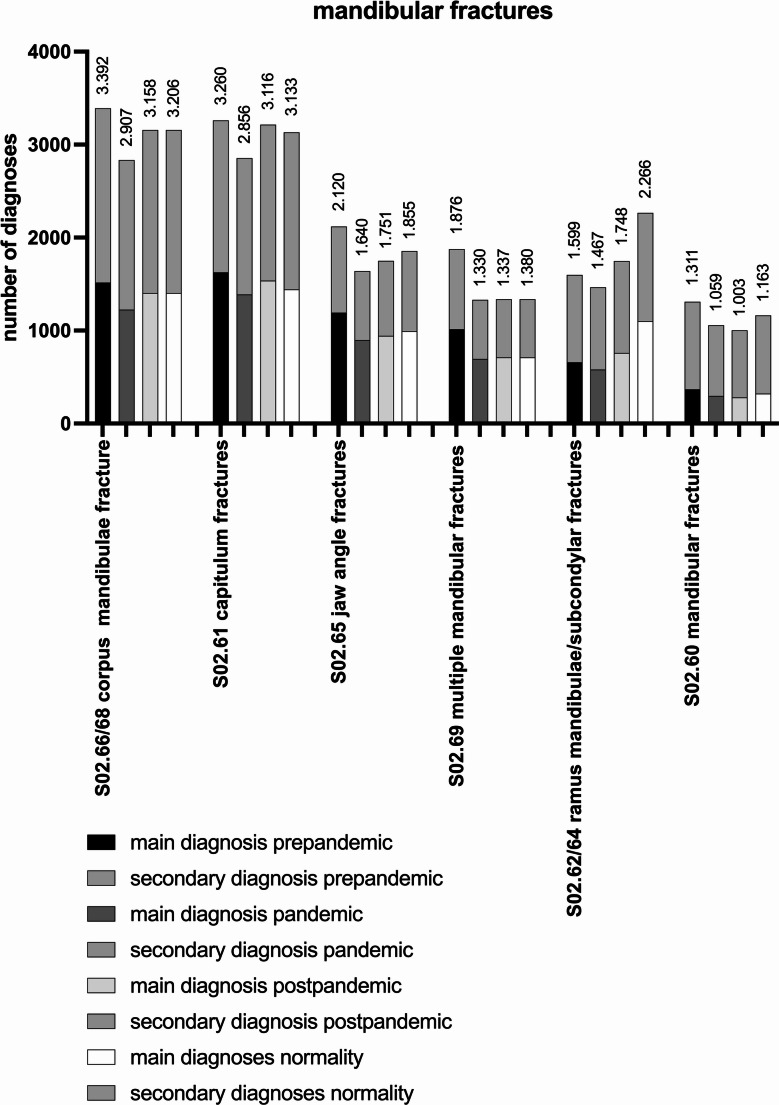
Number of inpatient diagnoses for mandibular fractures in the pre-pandemic, pandemic, post-pandemic period and normality

Concerning mandibular fractures and the pre-pandemic period, a total of 14.173 diagnoses (6. 548 MD, 7. 625 SD) was reported. In the pandemic period 11.904 (5.246 MD, 6.658 SD) diagnoses were recorded, which results in a decrease of 16.01% (*p* < 0.0001). Again, in the post-pandemic period 12.735 (5.780 MD, 6.955 SD) diagnoses were observed in total (+ 6.98%, *p* < 0.0001). In comparison between the pre-pandemic and post-pandemic period, a decrease of 10.15% (*p* < 0.0001) is recorded. During normality the total amount of 13.839 diagnoses (6.126 MD, 7.713 SD) is noted. Comparing the pre-pandemic period to normality, there is a decrease of 2.36% (*p* = 0,046). In comparison of the pandemic to normality there is an increase of 16.26% (*p* < 0.0001) and in comparison of post-pandemic to normality also an increase of 8.67% (*p* < 0.0001) occurred (Fig. 3).

Fractures of the corpus including the symphysis (S02.66/68) represented the most common fractures of the mandible: for the pre-pandemic period 3.392 diagnoses (1.517 MD, 1.875 SD) and 2.907 for the pandemic period (1.226 MD, 1.681 SD) were reported, which results in a decrease of 14.3% (*p* < 0.0001). In the post-pandemic period 3.158 diagnoses (1.405 MD, 1.753 SD) increased by 8.63% (*p* = 0.0013). In comparison between the pre-pandemic and post-pandemic period, also a decrease of 6.9% (*p* = 0.0038) is recorded.

During normality 3.206 diagnoses (1.424 MD, 1.782 SD) were noted. Pre-pandemic period in comparison to normality, there is a decrease of 5.48% (*p* = 0.022). In comparison of pandemic to normality there is an increase of 10,29% (*p* = 0.0001) and in comparison of post-pandemic to normality also an increase of 1.52% (*p* = 0.5474).

These fractures are followed by capitulum fractures (Code S02.61): 3.260 diagnoses (1.626 MD, 1.634 SD) pre-pandemic and 2.856 diagnoses (1.388 MD, 1.468 SD) in the pandemic period (−12.39%, *p* < 0.0001). In the post-pandemic period, 3.116 fractures were diagnosed. (1.537 MD, 1.579 SD). Comparing the post-pandemic with the pandemic period, an overall increment of 9,1% (*p* = 0.0008) is observed. In comparison between the pre-pandemic and post-pandemic period, also a decrease of 4.42% (*p* = 0.0714) is recorded.

During normality a total amount of 3.133 diagnoses (1.443 MD, 1.690 SD) was noted. Comparing the pre-pandemic period to normality, a decrease of −3,9% (*p* = 0.1122) was noted; in comparison of the pandemic to normality an increase of 9,70% (*p* = 0.0003) and in comparison of post-pandemic to normality also a increase of 0.55% (*p* = 0.8199) occurred.

The total amount of jaw angle fractures (S02.65) decreased by 22.64% (*p* < 0.0001) from 2.120 diagnoses (1.193 MD, 927 SD) in the pre-pandemic period and 1.640 diagnoses (897 MD, 743 SD) in the pandemic period. 1.751 (943 main diagnoses, 808 secondary diagnoses) were recorded in the post-pandemic period which resulted in an increase of 6.77% (*p* = 0.0567) in comparison to the pandemic period.

Comparing the pre-pandemic and post-pandemic period, a decrease of 17.41% (*p* < 0.0001) was recorded. During normality 1.855 diagnoses (993 MD, 862 SD) were noted. In comparison of the pre-pandemic to normality, a decrease of 12,50% (*p* < 0.0001) was observed. In comparison of the pandemic to normality, an increase of 13.11% (*p* = 0.0003) and comparing the post-pandemic to normality also an increase of 5.94% (*p* = 0.0833) was noted.

The number of multiple mandibular fractures (S02.69) decreased by 29.1% (*p* < 0.0001) from 1.876 diagnoses (1.015 MD, 861 SD) in the pre-pandemic period and 1.330 diagnoses (696 MD, 634 SD) in the pandemic period. Again, in the post-pandemic period 1.337 diagnoses (712 MD, 625 SD) occurred with an increase of 0.53% (*p* = 0.8922) in comparison to the pandemic period. Comparing pre-pandemic and post-pandemic period, a loss of 28.73% (*p* < 0.0001) was recorded. During normality 1.380 diagnoses (746 MD, 634 SD) were dated. Comparing the pre-pandemic period to normality a decrease of 26,44% (*p* < 0.0001) was observed. In comparison of the pandemic to normality, an increase of 3.76% (*p* = 0.3368) occurred and in comparison of post-pandemic to normality an increase of 3.22% (*p* = 0.4094) was observed.

Fractures of ramus mandibulae (S02.64) and subcondylar region (S02.62) counted for 1.599 diagnoses (658 MD, 941 SD) in the pre-pandemic period, 1.467 diagnoses (580 MD, 887 SD) in the pandemic period, respectively (−8.26%, *p* = 0.0172). Comparing the pandemic to the post-pandemic period, an overall increment of 19.15% (*p* < 0.0001) was noted from pandemic to post-pandemic period with a total of 1.748 fractures (760 MD, 988 SD). In comparison between the pre-pandemic and post-pandemic period, an increase of 9.32% (*p* = 0.01) was recorded. During normality 2.266 diagnoses (1.101 MD, 1.165 SD) were noted. Comparing the pre-pandemic period to normality there is an increase of 41.71% (*p* < 0.0001). In comparison of the pandemic to normality there is an increase of 54.46% (*p* < 0.0001) and in comparison of post-pandemic to normality also an increase of 29.63% (*p* < 0.0001) occurred.

Mandibular fractures without any specific classification (S02.60) have been evaluated with 1.311 diagnoses (369 MD, 942 SD) in the pre- and 1.059 diagnoses (296 MD, 763 SD) in the pandemic period (−19.22%, *p* < 0.0001). A total of 1.003 post-pandemic diagnoses (280 MD, 723 SD) was recorded (−5.29%, *p* = 0.2175). In comparison between the pre-pandemic and post-pandemic period, a decrease of 23.49% (*p* < 0.0001) was recorded. During normality 1.163 diagnoses (323 MD, 840 SD) were noted. Comparing the pre-pandemic period to normality there is a decrease of 11.29% (*p* = 0.0029). In comparison of the pandemic to normality there is an increase of 9.82% (*p* < 0.0001) and in comparison of the post-pandemic to normality also an increase of 15.95% (*p* = 0.0006) was noted (Table 2).

**Table 2:** List of the different alterations concerning the fractures of the mandible for the different time periods: pre-pandemic (pre), pandemic (pan), post-pandemic (post), normality (norm). Total numbers are given for the periods, percentage-based comparisons were conducted to evaluate changes across the respective groups (i.e. pre-pan, etc.)

		Total number	pre-pan (%)	pan-post (%)	pre-post (%)	pan-nor (%)	pre-nor (%)	post-nor (%)
Mandibular	Pre-pandemic	14.173						
	Pandemic	11.904	−16.01					
	Post-pandemic	12.735		6.98	−10.15			
	Normality	13.839				16.26	−2.36	8.67
Corpus	Pre-pandemic	3.392						
	Pandemic	2.907	−14.30					
	Post-pandemic	3.158		8.63	−6.90			
	Normality	3.206				10.29	−5.48	1.52
Capitulum	Pre-pandemic	3.260						
	Pandemic	2.856	−12.39					
	Post-pandemic	3.116		9.10	−4.42			
	Normality	3.133				9.70	−3.90	0.55
Jaw angle	Pre-pandemic	2.120						
	Pandemic	1.640	−22.64					
	Post-pandemic	1.751		6.77	−17.41			
	Normality	1.855				13.11	−12.50	5.94
Multiple	Pre-pandemic	1.876						
	Pandemic	1.330	−29.10					
	Post-pandemic	1.337		0.53	−28.73			
	Normality	1.380				3.76	−26.44	3.22
Ramus	Pre-pandemic	1.599						
	Pandemic	1.467	−8.26					
	Post-pandemic	1.748		19.15	9.32			
	Normality	2.266				54.46	41.71	29.63

### Midfacial fractures

**Fig. 4:**
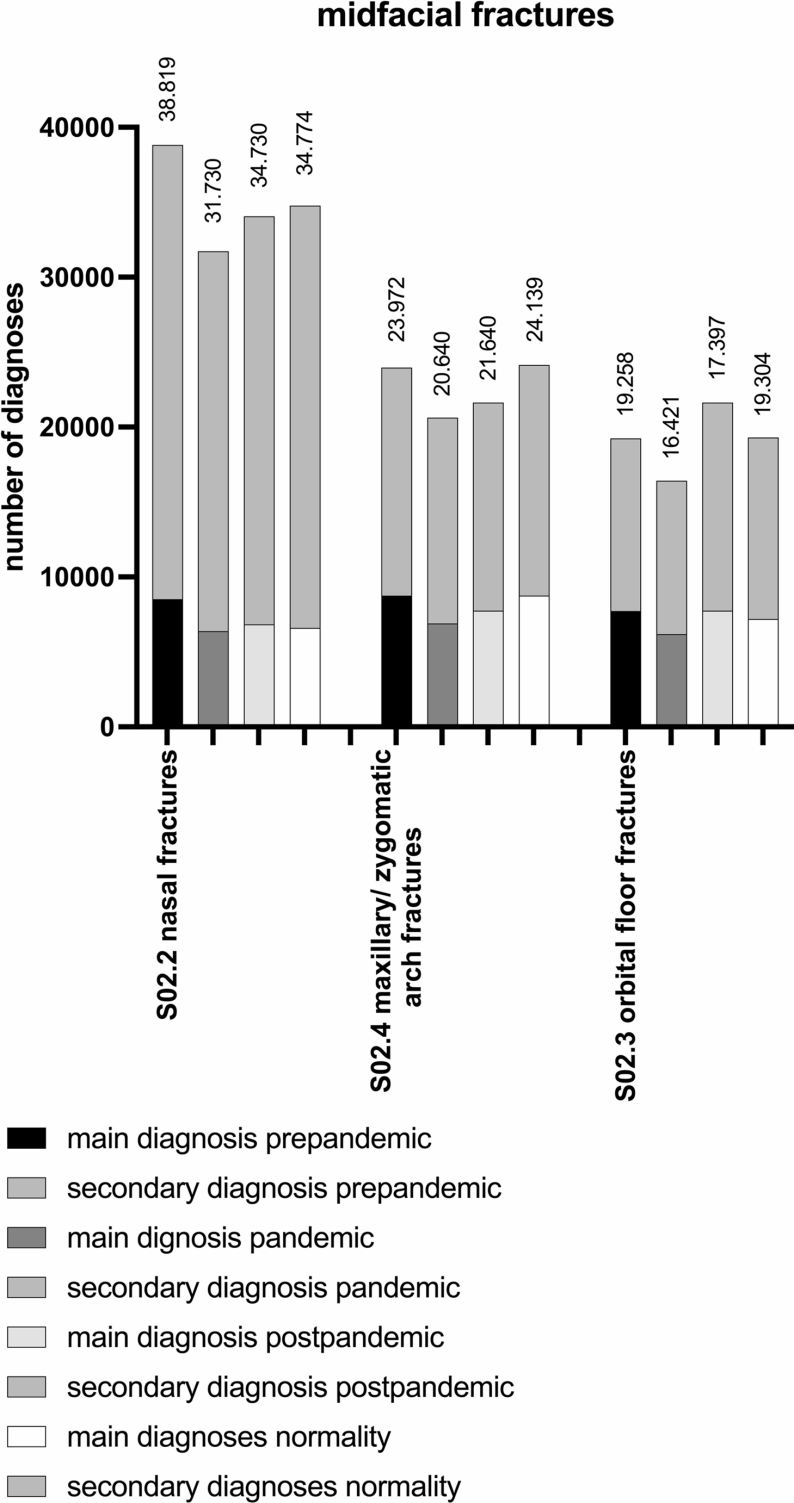
Number of inpatient diagnoses for midfacial fractures in the pre-pandemic, pandemic, post-pandemic period and normality

Midfacial fractures consisting of nasal bone fractures, orbital floor fractures, maxillary fractures and zygomatic arch fractures were represented in 82.049 cases (25.018 MD, 57.031 SD) in the pre-pandemic period. The number decreased to 68.791 diagnoses (19.482 MD, 49309 SD) in the pandemic period (−16.16%, *p* < 0.0001) (Fig. 4). The total amount of diagnoses during the post-pandemic period is 73.083 diagnoses (21.082 MD, 52.001 SD) (+ 6.24%, *p* < 0.0001). In comparison between the pre-pandemic and post-pandemic period, a decrease of 10.93% (*p* < 0.0001) was recorded.

During normality the total amount of diagnoses is 78.217 diagnoses (22.569 MD, 55.648 SD). Comparing the pre-pandemic period to normality, a decrease of 4.67% (*p* < 0.0001) was noted; in comparison of the pandemic to normality an increase of 13.70% (*p* < 0.0001) and in comparison of post-pandemic to normality an increase of 7.02% (*p* < 0.0001) occurred.

The most common fracture of the midface was the nasal bone fracture (S02.2) with 38.819 diagnoses (8.520 MD, 30.299 SD) in the pre-pandemic period and 31.730 diagnoses (6.392 MD, 25.338 SD) in the pandemic period, which corresponds to a decrease of 18.26% (*p* < 0.0001). In the post-pandemic period, 34.056 diagnoses (6.835 MD, 27.221 SD) were recorded (+ 7.33%, *p* < 0.0001). During normality 34.774 diagnoses (6.607 MD, 28.167 SD) were counted. Comparing the pre-pandemic period to normality, a decrease of 10.42% (*p* < 0.0001) was noted. In comparison of pandemic to normality, an increase of 9.59% (*p* < 0.0001) occurred and in comparison of the post-pandemic period to normality an increase of 2.11% (*p* = 0.0062).

Orbital floor fractures (S02.3) counted for 19.258 diagnoses (7.732 MD, 11.526 SD) in the pre-pandemic, 16.421 diagnoses (6.192 MD, 10.229 SD) in the pandemic period and 17.397 diagnoses (6.495 MD, 10.902 SD) in the post-pandemic period. Alterations are − 14.73% (*p* < 0.0001), + 5.94% (*p* < 0.0001) and − 9.66% (*p* < 0.0001), respectively. During normality the total amount of orbital floor fractures is 19.304 diagnoses (7.199 MD, 12.105 SD). Comparing the pre-pandemic period to normality there is an increase of 0.24% (*p* = 0.8148). In comparison of the pandemic to normality there is an increase of 17.56% (*p* < 0.0001) and in comparison of post-pandemic to normality also an increase of 10.96% (*p* < 0.0001).

The number of maxillary fractures and zygomatic arch fractures (S02.4) decreased by 13.9% (*p* < 0.0001) with 23.972 diagnoses (8.766 MD, 15.206 SD) in the pre-pandemic period to 20.640 diagnoses (6.898 MD, 13.742 SD) in the pandemic period. 21.630 (7.752 MD, 13.878 SD) were evaluated in the post-pandemic period leading to an increase of 4.8% (*p* < 0.0001) and decreased by 9.77% (*p* < 0.0001) for the comparison between the pre-pandemic and post-pandemic period, respectively. During normality 24.139 diagnoses (8.763 MD, 15.376 SD) were noted. Comparing the pre-pandemic period to normality an increase of 0.70% (*p* = 0.4464) was noted. In comparison of the pandemic to normality an increase of 11.6% (*p* < 0.0001) occurred and in comparison of the post-pandemic to normality an increase of 10.39% (*p* < 0.0001) (Table 3).

**Table 3:** Numbers and alterations between the periods (%) related to midfacial fractures

		Total number	pre-pan (%)	pan post (%)	pre-post (%)	pan-nor (%)	pre-nor (%)	post-nor (%)
Midface	Pre-pandemic	82.049						
	Pandemic	68.791	−16.16					
	Post-pandemic	73.083		6.24	−10.93			
	Normality	78.217				13.70	−4.67	7.02
Nasal	Pre-pandemic	38.819						
	Pandemic	31.730	−18.26					
	Post-pandemic	34.056		7.33	−12.27			
	Normality	34.774				9.59	−10.42	2.11
Orbital	Pre-pandemic	19.258						
	Pandemic	16.421	−14.73					
	Post-pandemic	17.397		5.94	−9.66			
	normality	19.304				17.56	0.24	10.96
Maxillary	Pre-pandemic	23.972						
	Pandemic	20.640	−13.90					
	Post-pandemic	21.630		4.80	−9.77			
	Normality	24.139				16.95	0.70	11.60
Cranial	Pre-pandemic	22.398						
	Pandemic	20.535	−8.32					
	Post-pandemic	20.420		−0.56	−8.83			
	Normality	21.983				7.05	−1.85	7.65
Scull base	Pre-pandemic	14.559						
	Pandemic	13.588	−6.67					
	Post-pandemic	13.312		−2.03	−8.57			
	Normality	14.460				6.42	−0.68	8.62

### Other fractures including fractures of the skull base

The number of cranial and facial bone fractures decreased in the pandemic period compared to the pre-pandemic period: 22.398 diagnoses (4.802 MD, 17.596 SD) count for the group of cranial and facial bone fractures in the pre-pandemic period, 20.535 diagnoses (4.289 MD, 16.246 SD) for the pandemic (−8.32%, *p* < 0.0001) and, 20.420 (4.158 MD, 16.262) in the post-pandemic period (−0.56%, *p* = 0.5699). In comparison between the pre-pandemic and post-pandemic period, a decrease of 8.83% (*p* < 0.0001) was recorded. During normality 21.983 (4.233 MD, 17.750 SD) of cranial and facial bone fractures were noted. Comparing the pre-pandemic period to normality a decrease of 1.85% (*p* = 0.0489) was observed. In comparison of the pandemic to normality an increase of 7.05% (*p* < 0.0001) occurred and in comparison of the post-pandemic to normality an increase of 7.65% (*p* < 0.0001).

The total amount of scull base fractures (S02.1) was recorded with 14.559 diagnoses (3.213 MD, 11.346 SD) in the pre-pandemic and 13.588 diagnoses (2.927 MD, 10.661 SD) in the pandemic period, which results in decrease of 6.67% (*p* < 0.0001). The number of diagnoses during the post-pandemic period is 13.312 diagnoses (2.802 MD, 10.510 SD). The overall decrease is 2.03% (*p* = 0.0924) in comparison to the pandemic period. In comparison between the pre-pandemic and post-pandemic period, a decrease of 8.57% (*p* < 0.0001) was recorded. During normality 14.460 diagnoses (2.918 MD, 11.542 SD) were dated. In comparison of the pre-pandemic period to normality a decrease of 0.68% (*p* = 0.5611) occurred. In comparison of the pandemic to normality an increase of 6.42% (*p* < 0.0001) was observed and in comparison of post-pandemic to normality an increase of 8.62% (*p* < 0.0001).

**Fig. 5:**
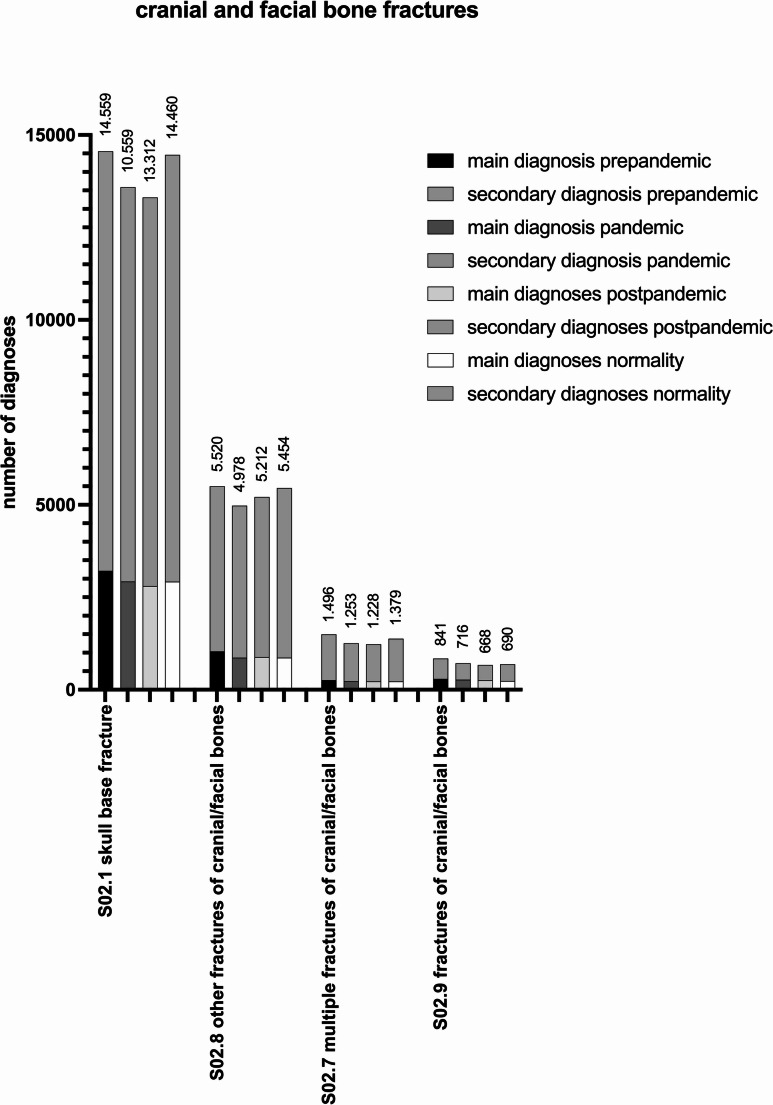
Number of inpatient diagnoses for cranial and facial bone fractures in the pre-pandemic, pandemic, post-pandemic period and normality

Figure 5 reveals other fractures related to the cranial bones.

## Discussion

For the first time, this retrospective study evaluated the frequency patterns in inpatients with CMF fractures in times and after COVID-19 by official governmental data in Germany. Therefor data of the InEK-Institute has been evaluated with the use of ICD-10 coding system: due to COVID-19, presentations in the emergency departments were significantly reduced by 14.66% for all cases (MD and SD) in comparison between the pre- and pandemic period. Again, in the post-pandemic the frequency raised by 4.95%. Still, comparing the pre- and post-pandemic period a decrease of 10.44% was noted.

Nasal bone fractures (S02.2) are most common with 38.819 total diagnoses in the pre-pandemic period, 31.730 diagnoses during the pandemic, 34.065 diagnoses during the post-pandemic period and 34.774 during normality.

The underlying study confirms the decline of CMF cases in Germany in general, going in line with a worldwide decrease in case presentations due to COVID-19 for surgical entities [3, 4]. This change may be dependent on several causes and shows differences between distinguished surgical and non-surgical specialties. For example, studies show similar decrease in other non-surgical departments like Ear nose throat (ENT) or hematooncology [5, 6]. In contrast, the number of radial fractures slightly differs between the 4 time periods. The origin might be found in the trauma mechanism such as high energy trauma associated with CMF fractures, and radius fractures often associated with low falls in elderly patients. In Germany, most CMF trauma cases initially present in outpatient emergency settings, with only moderate to severe injuries – such as fractures requiring surgical intervention – being admitted as inpatients. Approximately a low amount of all CMF trauma patients require inpatient treatment, whereas the remaining cases, including isolated nasal or minor midfacial fractures, are typically managed on an outpatient basis. This context helps classify the proportions observed in our dataset, which exclusively reflects inpatient cases.

CMF trauma is mainly caused by high energy mechanisms and forces in daily life, such as traffic accidents, falls, sports accidents, work accidents or domestic violence. Khan et al. recently reported on the hierarchy of traffic accidents followed by violence, falls and sports accidents [7]. Viozzi et al. evaluated the influence of different sport disciplines on the occurrence of maxillofacial fractures: football, soccer games, hockey and baseball most often count for the sports-related injuries in combination with fractures of the head [8]. Jin et al. evaluated the origin of fractures of the mandible, they found the main reason for mandibular angle fractures is violence with 41.6%, followed by ground accident 37.6%. Concerning fractures of the midface, they reported zygomatic arch and maxillary fractures most commonly resulted by ground accidents 51.1%, followed by traffic accidents 22.8% [9]. Also, high-level falls represent a critical mechanism, especially in the context of traumatic brain injury [10]. In general, falls represent the most common mechanism of injury in patients presenting to trauma emergency departments [11].

Accordingly, due to the closing of public spots, restaurants, bars, entertainment establishments, travel restrictions and the cancellation of sporting events, the risk for CMF injury is lowered. Concerning other European countries like Italy, COVID-19 entails a decline in case presentations of maxillofacial fractures and other trauma categories as less traffic accidents are noticed and sport events were cancelled. Vishal et al. approved this by reporting a decrease of maxillofacial trauma during COVID-19-lockdown in India [12]. The study allocated trauma origins to traffic accidents, physical assault, fall and animal attack. A decline in traffic movement led to a reduction of traffic accidents and resulted in less maxillofacial trauma case presentations.

Even before the worldwide boost of remote working and home office within the COVID-19, Soltani et al. showed in an Australian retrospective study a reduction of work accidents when companies allowing home office, factories and schools are closed [13]. Beak et al. also attest a reduction in work accidents in a comparison study between 2016 and 2020 concerning COVID-19 [14].

Nevertheless, isolation and domestic issues risen in a lockdown period can cause a higher rate of assaults [15–17]. Fractures which are caused by domestic violence may have increased due to a difficult domestic situation during lockdown. Vishal et al. evaluated an increase of physical assault by 50% [12]. Especially intimate partner violence and domestic violence increased in Spain, China, Brazil and France [18]. This issue is also discussed in general newspaper writing not only in Germany that aggression in public environment is decreasing while domestic violence is raising in a period of a public lockdown [19].

The underlying study also confirmed the hypothesis that CMF surgery case presentations increased again in the post-pandemic period and again during normality; still ongoing as a gradual return to normal life. Comparing the pre-pandemic and post-pandemic period, the number of fractures is reduced in the post-pandemic period compared to the pre-pandemic period (exception Code S02.2/62/64). This may be caused by a highered level of awareness and changes in lifestyle influenced by the pandemic [20]. Along this, Campagnoli et al. noted that people refrained from hospitals in case of minor trauma to avoiding infections in the pandemic [6]. In general, the reduction of CMF surgery cases might find the origin in the reduced interaction between patients and health care system to a certain amount in the pandemic and the post-pandemic period. Additionally, altered health-seeking behavios - such as avoiding medical facilities for minor injuries - could have contributed to an underreporting of less severe nasal trauma. At present, the literature lacks definitive data on this observation within the field of maxillofacial surgery.

Next to case presentations, also elective operations have been postponed due to COVID-19 and pandemic-related restrictions [21, 22]. This trend during a global pandemic raises the question of resource allocation especially concerning CMF surgery as a finite resource.

To ensure an efficient use of resources and time, care for all emergency patients, redistribution concepts for all belongings of a health care system should be discussed. Recently, Ndayishimiye et al. report in a literature review on short and long term strategies including repurposing established buildings, remote adjustments and building new structures, respectively [23]. The authors mainly focus on infrastructure and not medical staff. Semenova et al. documented the pandemic period in 2020 in the less developed health care system of Kazakhstan and stress the importance of allocation medical personal and training for hygiene and the use of extracorporeal membrane oxygenation (ECMO) devices [24]. Next to adjustment mechanism and allocation of medical staff in an acute crisis of a health care system, the authors ask for general guidelines and prevention of spread.

COVID-19 puts also high financial risk on health care providers. However, employers do not obtain a special termination right in times of low income and underemployment [25]. Therefore, medical staff set free due to caring for a lower number of emergency patients during pandemic times should be used as a resource and back-up plan for the emergency department or intensive care units. During COVID-19, also doctors in private practices have been recruited to support colleagues, especially for vaccination [26]. This concept might be obtained in the post-pandemic period as well. It is possible that further medical training which is specifically aimed at intensive care and emergency medicine could improve the redistribution of internal medicine resources in exceptional cases. In the particular case of CMF surgery, the training requires long time and a wide variety of learning contents. Deductively, a CMF resident is a specific, valuable and rare working force which should be present in a longterm contract and working condition. All concentrations on a stable working relation should be paid also in times of the pandemic with the intention not to lose employees.

The analyzed data is lacking: The data utilized in this study were of a retrospective and secondary nature, collected and structured by institutional processes in a manner representative of the principles outlined in the Declaration of Helsinki. There is heightened attention to accurate documentation due to its direct impact on hospital funding. Additionally, it is important to note that no prehospital information, such as the origin of the trauma or fracture, is captured in this data set. No data about mortality or treatment options (conservative vs. intervention/surgery) is given.

In addition, due to data curation, the absolute patient number is reflected only in the main diagnosis. The given frequencies depend on the sum of the main and secondary diagnoses. Searching for a distinctive ICD-10 Code, a main and secondary diagnosis are given. The total number of all patients was mentioned, which reflects only the number of patients included in the group of main diagnoses as one diagnosis counts for one patient. No data.

Since the InEK database does not permit the derivation of corresponding primary diagnoses from secondary diagnoses, the classification of injury patterns remains limited. To better contextualize the frequency of injuries in polytraumatized patients, data from the annual report of the German Trauma Registry (DGU TR 2021) were consulted. This report includes a total of 88,327 patients documented between 2019 and 2021. Among them, 45.5% (*n* = 40,242) sustained head injuries, 10.6% (*n* = 9,320) facial injuries, 45.2% (*n* = 39,940) thoracic trauma, and 13.9% (*n* = 12,315) injuries to the abdominal region – reflecting that only a smaller proportion of patients with craniomaxillofacial (CMF) trauma sustain injuries of sufficient severity to warrant inclusion in the trauma registry [27].

## Conclusion

Facial trauma and consecutive hospital admissions have been decreased after the beginning of COVID-19 and have been overall increased during post-pandemic times. Still, the pre-pandemic frequency is not yet reached.

## Data Availability

No datasets were generated or analysed during the current study.
